# Real-Time Carbon Content Prediction Model for the Reblowing Stage of Converter Based on PI-LSTM

**DOI:** 10.3390/ma18194631

**Published:** 2025-10-08

**Authors:** Yuanzheng Guo, Dongfeng He, Xiaolong Li, Kai Feng

**Affiliations:** 1 State Key Laboratory of Advanced Metallurgy, University of Science and Technology Beijing, Beijing 100083, China; 15100570223@163.com (Y.G.); lxlustb@outlook.com (X.L.); fengkai-show@163.com (K.F.); 2 Department of Ferrous Metallurgy, School of Metallurgical and Ecological Engineering, University of Science and Technology Beijing, Beijing 100083, China

**Keywords:** steel reablowing stage of converter, carbon content, PI-LSTM, real-time prediction

## Abstract

Precise forecasting of carbon content in the converter’s reblowing phase is pivotal to boosting steel production efficiency and ensuring effective control over molten steel quality. However, existing mechanistic models based on material balance and decarbonization kinetics suffer from insufficient accuracy due to simplifying assumptions. In contrast, data-driven models rely on data quality, lack generalization capability, and lack physical interpretability. Additionally, integral models based on flue gas analysis suffer from data latency issues. To overcome these limitations, this study proposed a real-time carbon content prediction model for the converter’s reblowing phase, leveraging a physics-informed long short-term memory (PI-LSTM) network. First, flue gas data was processed using a carbon integration model to generate a carbon content change curve during the reblowing stage as a reference for actual values; second, a dual-branch network structure was designed, where the LSTM branch simultaneously predicts carbon content and key unmeasurable parameters in the decarbonization kinetics, while the mechanism branch combined these parameters with the decarbonization formula to calculate carbon content under mechanism constraints; finally, a joint loss function (combining data-driven loss and mechanism constraint loss) was used to train the model, and the gray wolf optimization (GWO) algorithm was employed to optimize hyperparameters. Experimental results show that compared to the mechanism model (MM) and LSTM model, the PI-LSTM model achieves an average absolute error (MAE) of 0.0077, a root mean square error (RMSE) of 0.0112, and endpoint carbon content hit rates within ±0.005%, ±0.01%, ±0.015% error ranges, achieving 53.71%, 82.23%, and 95.45%, respectively, significantly improving prediction accuracy and physical plausibility. This model lays a robust groundwork for dynamic closed-loop real-time control of carbon levels in the converter’s reblowing stage.

## 1. Introduction

Converter steelmaking involves top oxygen injection via lances and bottom gas agitation to lower carbon content and harmful elements (e.g., phosphorus, sulfur) in molten iron, scrap, and slag formers (such as dolomite, lime) to meet grade-specific standards. It also adjusts molten steel temperature and carbon levels to align with tapping specifications [1]. Converter steelmaking is an exceedingly intricate, high-temperature physicochemical process characterized by turbulent multiphase flow and chemical reactions [2]. Additionally, in high-temperature, corrosive chemical environments, existing detection methods cannot achieve continuous monitoring of the composition and temperature within the melt pool. As a result, most steel mills currently rely on manual experience for converter endpoint control, resulting in low control accuracy and instability. Being a key procedure, accurate regulation of endpoint carbon levels not only avoids excessive oxidation of molten steel and reduces post-furnace alloy consumption, but also aids in lowering carbon emissions in steelmaking. This, in turn, facilitates high-quality, efficient, and stable operations in converter smelting.

Currently, the primary methods for real-time prediction of carbon content in converters include MMs [3], data-driven models [4], and hybrid models [5]. The mechanistic model is primarily based on material balance and decarbonization kinetics. Dering et al. [6] proposed a first-principles-based dynamic model for the basic oxygen furnace (BOF), which comprehensively considers the major physical and chemical processes involved in the converter, including slag formation mechanisms and thermal equilibrium relationships. This model ultimately constructs a system of differential algebraic equations (DAEs) capable of predicting the decarbonization reaction process in the BOF. According to Root et al., [7] based on multi-zone reaction kinetics, the decarbonization process in a converter is primarily divided into a jet impact zone and a slag-metal emulsification zone. Considering multiple factors, a corrected mass transfer coefficient and droplet size distribution function (RRS distribution) are introduced to calculate the total decarbonization rate by adjusting the droplet generation rate. Wang et al. [8], based on the three-stage decarbonization theory and considering time-series data, use an explicit finite difference method to establish a continuous carbon content prediction model to achieve real-time prediction of carbon content. Although the above mechanism model is highly interpretable, it involves many key parameters that cannot be directly measured or calculated. It introduces many assumptions and simplifications, resulting in a low model accuracy.

Alongside the swift advancement of computer technology, networking technologies, artificial intelligence, and big data technologies, data-driven models are now widely used for predicting the carbon content endpoint in converters [9,10,11]. Real-time carbon content prediction based on data-driven methods primarily relies on real-time detection techniques such as converter flue gas analysis and converter flame imaging. Such approaches are not reliant on in-depth comprehension of a system’s inherent physical mechanisms; instead, they emphasize extracting patterns from and analyzing the data itself. Given that flue gas sensing devices are positioned remotely from the molten bath reaction area, the resulting flue gas data exhibit a measurable time lag. To address this issue, Gu et al. [12] fitted the real-time changes in converter carbon content using historical flue gas data. They established a dynamic carbon level forecasting model for the converter’s reblowing phase, leveraging case-based reasoning integrated with long short-term memory neural networks (CBR-LSTM) and utilizing time-series data as input. The flame patterns in steelmaking converter exhibit multi-directional, multi-scale irregular characteristics, making them difficult to describe. Liu et al. [13] developed a flame image feature extraction approach leveraging a derivative-driven nonlinear mapping direction-weighted multi-layer complex network (DDMCN), integrated with a KNN regression model to enable real-time forecasting of carbon content in converter smelting. Data-driven approaches can uncover hidden patterns and correlations in data, though their accuracy is significantly dependent on the quality and quantity of the data [14,15]. Additionally, the lack of mechanistic knowledge may lead to violations of physical laws such as the conservation of mass.

To address the limitations of mechanistic models, which suffer from prediction biases due to simplifying assumptions, and data models, which lack physical interpretability, a hybrid model integrating data-driven and mechanistic approaches has been developed. This model leverages mechanistic knowledge to constrain model structure and reduce reliance on data, while utilizing data-driven methods to capture complex nonlinear relationships [16]. Li et al. [17], based on furnace gas analysis data, introduced melt pool homogeneity into the traditional exponential model, and used nonlinear fitting to calculate the impact of time-series data on the decarbonization rate of the melt pool. Integrating this with slag molecular theory, they developed a hybrid model for real-time forecasting of carbon levels in converter operations. However, the core of the model still relies on predefined functions and offline parameter calibration, making it challenging to respond in real time to the dynamic effects of instantaneous operations such as gun position changes and flow fluctuations. To enhance adaptability to dynamic processes, Wang et al. [18] converted the conventional time-dependent exponential decarburization kinetic model into an oxygen-centric form. They employed case-based reasoning (CBR) to determine key model parameters, developed a hybrid model, and thereby enabled real-time forecasting of carbon levels in the converter’s reblowing phase. While this method improves parameter adaptability through data-driven approaches, it lacks direct constraints on the core principles of decarburization kinetics, making it challenging to avoid potential physical logic biases introduced by data-driven methods. To further strengthen mechanism constraints, Xia et al. [19] developed a physical information neural network (PINN)-based model for predicting the final carbon content of a converter, achieving a hit rate of 93.52% within an error range of ±0.02%. PINN embeds physical laws directly into the neural network’s loss function, allowing the model to capture data correlations while abiding by predefined physical constraints [20]. However, the model’s static network structure fails to capture dynamic variations in time-series data [21], restricting its use to forecasting endpoint carbon content in the blowing process instead of continuously monitoring carbon content changes throughout the process [22].

Under the PINN framework, traditional neural networks are substituted with LSTM [23], resulting in the formation of a physics-informed long short-term memory neural network (PI-LSTM) [24]. This PI-LSTM hybrid model has shown notable practical utility across various domains, including wave forecasting, material property prediction, and motor stator evaluation [25,26,27,28]. Still, no studies have yet been reported on its use for real-time prediction of carbon content in converters. This approach integrates the physical law compliance of PINNs with LSTM’s ability to handle nonlinear time series [29], rendering it well-suited for dynamic modeling in converter carbon content forecasting. In contrast to other hybrid modeling techniques, PI-LSTM inherently embeds physical constraints, lessening reliance on data and boosting the model’s generalizability and interpretability [30]. In contrast to PINN, the gating mechanism within PI-LSTM precisely captures the temporal characteristics of how variations in lance position and flow rate during the converter’s reblowing phase influence fluctuations in carbon levels. To conclude, through integrating time-series data characteristics of converter smelting with decarbonization dynamics, PI-LSTM can tackle the intricate dynamic properties of converter decarbonization modeling.

This study addresses the issues of intense data lag in blast furnace blowing process flue gas data and the limitations of current real-time carbon content prediction models. It innovatively constructs a real-time carbon content prediction model targeting the reblowing phase of blast furnaces, leveraging PI-LSTM. First, historical flue gas data from the converter were subjected to time-series alignment processing. Based on the concentrations of CO and CO_2_ in the exhaust gas, a carbon integration model was used to fit the carbon content change curves for each historical furnace run during the reblowing stage, serving as the calibration benchmark for the actual carbon content values. Second, based on an analysis of the decarbonization kinetics mechanism, the study focused on ascertaining non-measurable parameters in the decarbonization equation during the final stage of the converter procedure. The model employed a dual-branch network structure of LSTM models to simultaneously predict data-driven carbon content and unmeasurable parameters in the mechanistic formula. It used a dual loss function combining data-driven loss and physical mechanism constraint loss, training the model with process data as input and fitted carbon content change curves as output values. The model hyperparameters were optimized using the gray wolf algorithm. Eventually, a real-time carbon content forecasting model for the converter’s reblowing stage leveraging PI-LSTM is constructed, offering a basis for accurate closed-loop control of carbon levels in the converter blowing process.

## 2. Analysis of the Converter Steelmaking Process

### 2.1. Converter Smelting Process

**Figure 1:**
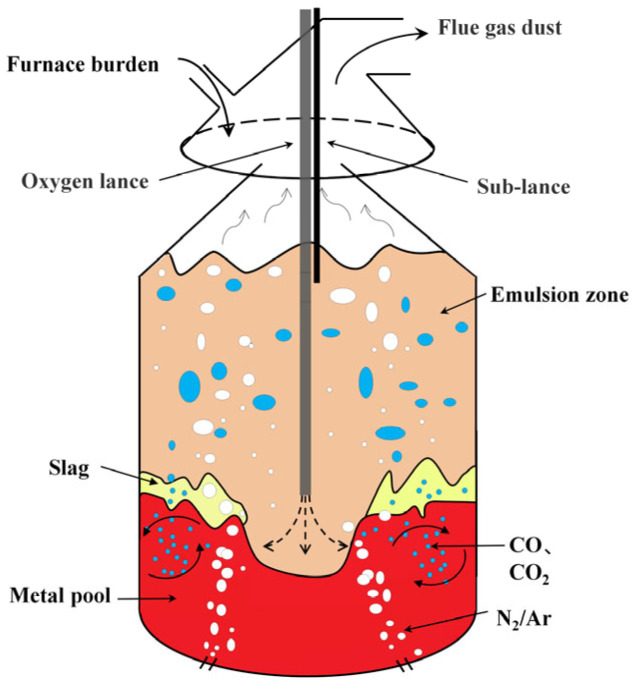
Converter diagram.

As a high-temperature, high-pressure metallurgical reactor, the converter undergoes intense multi-component, multi-phase reactions [31], as shown in Figure 1. The objective of smelting is to reduce impurity elements in molten iron to meet specified thresholds and increase its temperature from 1350 °C to around 1650 °C [32]. According to the operation time of the auxiliary lance, the blowing process is categorized into the main blowing stage and the reblowing stage. In the main blowing stage, scrap steel and molten iron are charged into the converter in sequence. Oxygen is subsequently injected into the molten bath through the oxygen lance at a defined flow rate, while auxiliary fluxes including lime and calcined dolomite are fed in batches. When the oxygen supply reaches approximately 85%, the auxiliary lance is inserted to measure the carbon content and temperature of the melt pool (TSC); In the secondary blowing stage, the required oxygen supply or cooling agent addition is adjusted based on the TSC measurement. After blowing is completed, the auxiliary lance is reinserted to measure the carbon content and temperature (TSO). If TSO meets the endpoint control requirements, alloying is performed; otherwise, re-blowing is conducted [33].

### 2.2. Converter Decarbonization Process

**Figure 2:**
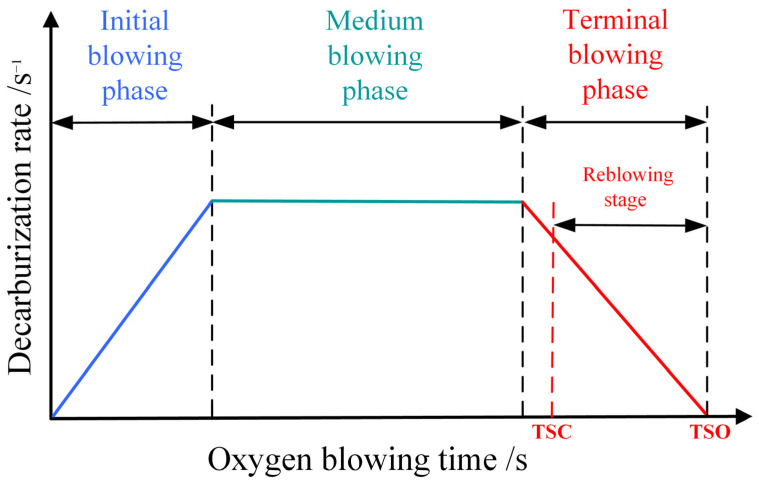
Traditional three-stage decarburization for BOF process.

Based on the stage-specific characteristics of decarburization kinetics during the converter blowing process, the decarburization process falls into three phases: the initial phase, the middle phase, and the terminal phase of blowing [34], as illustrated in Figure 2. During the initial phase of blowing, the melt pool temperature is relatively low (typically < 1400 °C), and oxygen preferentially oxidizes the silicon and manganese elements in the molten iron, while the carbon-oxygen reaction is inhibited. The decarburization rate is gradual and exhibits linear growth [35]. During the medium blowing phase, as silicon and manganese content significantly decrease and the melt pool temperature rises above 1500 °C, the decarburization reaction enters a high-speed stable phase, reaching a peak decarburization rate that remains constant. At this stage, the rate is primarily governed by oxygen supply intensity, meaning that the higher the oxygen flow rate, the higher the decarburization rate. In the terminal blowing phase, once the carbon content drops below the critical value, the limiting factor of the decarburization reaction shifts to carbon mass transfer and diffusion, and the decarburization rate decreases exponentially with the reduction in carbon concentration. At this point, even if the oxygen supply is increased, the oxygen utilization rate decreases, leading to an exacerbation of oxygen accumulation in the melt pool [8].

### 2.3. Converter Flue Gas Analysis

During the converter smelting process, CO generated by the decarburization reaction undergoes secondary combustion within the furnace, with part of it oxidizing into CO_2_, forming converter furnace gas primarily composed of CO and CO_2_. The converter furnace gas rises to the hood, where it mixes with air drawn in from outside the hood and undergoes secondary combustion outside the furnace, forming converter flue gas primarily composed of CO, CO_2_, O_2_, N_2_, Ar, H_2_, and other gases. Converter flue gas analysis and dynamic control continuously monitor the composition and flow rate of flue gas generated during the blowing process. Through model calculations, it provides real-time online forecasts of steel melt element composition and temperature changes, issues warnings, and controls slag conditions. It adjusts oxygen supply flow rates and slag-making regimes online to improve steel melt quality and final target achievement rates.

**Figure 3:**
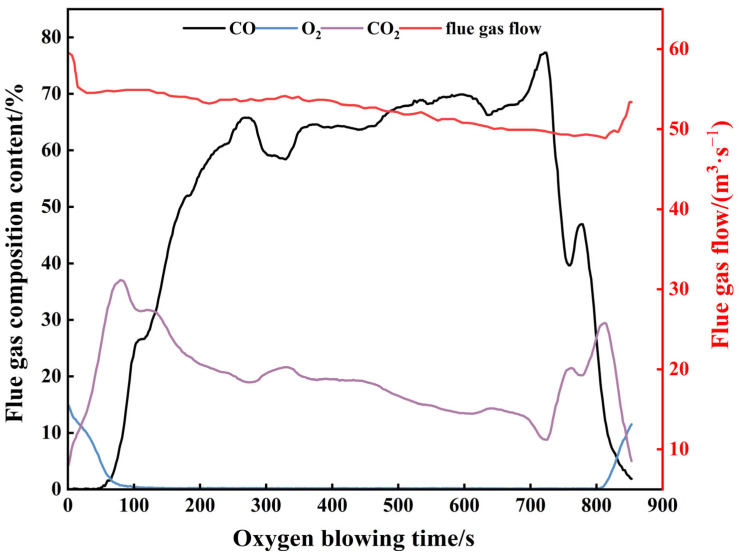
Flue gas flow rate and volume fraction of main gases.

Key components of the flue gas and variations in flue gas flow rate in the converter smelting process are illustrated in Figure 3. During converter smelting, the trends of CO and CO_2_ are opposite. At the end of smelting, fluctuations in CO and CO_2_ content are the result of sampling using an auxiliary lance. This is because after the auxiliary lance is inserted, the oxygen lance stops supplying oxygen, causing the CO combustion rate to slow down and resulting in a slight increase in CO content. The trend of CO changes in the figure generally aligns with the three-stage decarbonization theory, as the proportion of CO generated by carbon-oxygen reactions in the furnace smelting process is very high in the flue gas [36]. By virtue of the volume fractions of CO and CO_2_ in flue gas and its flow rate, the decarbonization rate in the melt pool can be computed via the carbon balance during converter smelting (Equation (1)).

(1) $$v_{w}=\frac{12}{22.4}\times Q_{smoke}\times [\varphi (CO)+\phi (CO_{2})]$$

In Equation (1), $v_{w}$ represents the decarbonization rate, kg/s; $Q_{smoke}$ represents the flue gas flow rate, Nm^3^/s; φ(CO), $\varphi (CO_{2})$ represent the volume fractions of CO and CO_2_ in the flue gas.

The carbon credit model enables the calculation of the molten pool’s carbon content by deducting the quantity of carbon continuously released from furnace gas as CO and CO_2_ from the total carbon content in the TSC molten pool. The residual quantity corresponds to the carbon content in the molten steel pool [17]. The molten pool instantaneous carbon $y_{1}(t)$ content can be obtained solving Equation (2):

(2) $$y_{1}(t)=(y-\int_{0}^{t}v_{w}dt{)/W}_{st}$$

In Equation (2), $y_{1}(t)$ represents the carbon content at time t, %; y represents the total carbon content under initial conditions, kg; represents the molten steel mass, kg.

Currently, on-site carbon content measurements are only available at the TSC and TSO time points, with no data available for carbon content during the process. Although, flue gas data inherently exhibits lag due to the influence of flue gas transmission pathways and the response characteristics of detection equipment, resulting in delays during online smelting, it is possible to align flue gas data with non-delayed sequential data, such as oxygen flow rates, based on the delay time. This allows the carbon content during the historical data two-blow process be calculated using a carbon integration model.

## 3. Model Principle

### 3.1. Decarbonization Kinetics During the Reblowing Stage of a Converter

Tao et al. [37] proposed a decarburization kinetic equation for the transition from TSC to TSO in steelmaking converter based on the research on dynamic control of the auxiliary gun by Robertson et al. [38]:

(3) $$\frac{\left[x_{1}(t)-x_{1}\left(t_{0}\right)\right]+h_{0}\cdot \left[x_{2}(t)-x_{2}\left(t_{0}\right)\right]}{W_{steel}}=\frac{\beta_{m}}{\alpha_{m}}\cdot \ln\left\{\frac{\exp\left[\frac{C_{TSC}-C_{0}}{\beta_{m}}\right]-1}{\exp\left[\frac{y_{1}(t)-C_{0}}{\beta_{m}}\right]-1}\times \frac{\exp\left[-\frac{C_{TSC}-C_{0}}{\beta_{m}}\right]}{\exp\left[-\frac{y_{1}(t)-C_{0}}{\beta_{m}}\right]}\right\}$$

In Equation (3), *x*_1_(*t*) and *x*_1_(*t*_0_) represent the oxygen blowing rate at the current time *t* and the TSC measurement time *t*_0_, respectively, in Nm^3^. *x*_2_(*t*) and *x*_2_(*t*_0_) represent the coolant weight at times *t* and *t*_0_, respectively, in t. *W_steel_* is the molten steel weight, in t. C*_TSC_* is the carbon content measured by TSC, in %. C_0_ is the critical carbon content, in %. The critical carbon content is typically related to the stirring intensity of the melt pool and ranges between 0.2% and 0.6% [8]. *h_0_* sents the oxygen supply per unit weight of coolant, in Nm^3^/t. *y*_1_(*t*) represents the carbon content of the molten steel at the current time *t*, in %. *α_m_* represents the parameter related to the decarburization reaction, in t/Nm^3^. It reflects the intensity of the influence of oxygen injection on the decarburization rate, with a range of [1, 2]. *β_m_* is the kinetic characteristic parameter of the decarburization reaction, related to the carbon content C*_TSC_* and critical carbon content C_0_, with a range of [20, 40].

By transforming Equation (3), the carbon content of molten steel can be calculated:

(4) $$y_{1}(t)=C_{0}+\beta_{m}\ln\left\{1+\left[\exp\left(\frac{C_{TSC}-C_{0}}{\beta_{m}}\right)-1\right]\cdot \exp\left[\frac{\alpha_{m}}{\beta_{m}}\cdot \frac{\left[x_{1}(t)-x_{1}\left(t_{0}\right)\right]+h_{0}\cdot \left[x_{2}(t)-x_{2}\left(t_{0}\right)\right]}{W_{steel}}\right]\right\}$$

During the reblowing control phase of the converter, the coolant is mainly used for cooling, and its oxygen supply can be disregarded in terms of its effect on decarburization. When the oxygen supply of the coolant is ignored, Equation (4) can be simplified to Equation (5):

(5) $$y_{1}(t)=C_{0}+\beta_{m}\ln\left\{1+\left[\exp\left(\frac{C_{TSC}-C_{0}}{\beta_{m}}\right)-1\right]\cdot \exp\left[-\frac{10\alpha_{m}}{\beta_{m}}\left(\frac{x_{1}(t)-x_{1}\left(t_{0}\right)}{W_{steel}}\right)\right]\right\}$$

In Equation (5), $x_{1}(t)-x_{1}\left(t_{0}\right)$ represents the cumulative oxygen consumption $V_{O_{2}}$ from the TSC time to the current time, in Nm^3^, as shown in Equation (6):

(6) $$y_{1}(t)=C_{0}+\beta_{m}\ln\left\{1+\left[\exp\left(\frac{C_{TSC}-C_{0}}{\beta_{m}}\right)-1\right]\cdot \exp\left[-\frac{10\alpha_{m}}{\beta_{m}}\left(\frac{V_{O_{2}}}{W_{steel}}\right)\right]\right\}$$

### 3.2. Physical Information Long-Term and Short-Term Memory Neural Network

#### 3.2.1. LSTM

**Figure 4:**
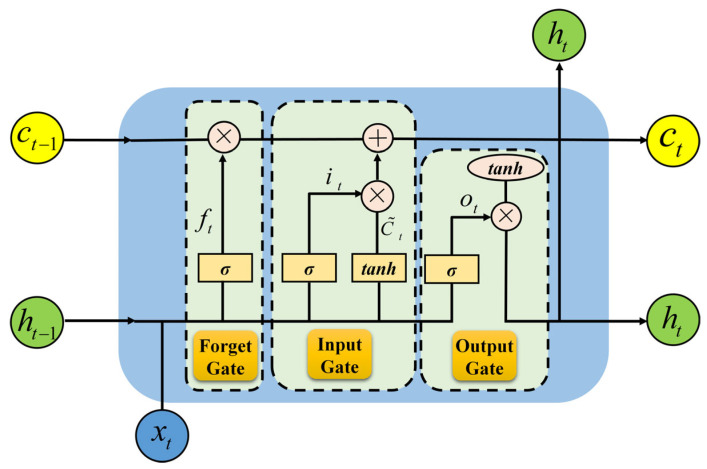
LSTM internal structure.

LSTM represents a specific category of recurrent neural network architecture, capable of addressing the gradient vanishing issue existing in conventional recurrent neural networks (RNNs), thus enabling the capture of long- and short-term dependences within time series data [39,40]. The core structure of LSTM integrates three gate control mechanisms and a storage unit to achieve selective storage and forgetting of information [41], as shown in Figure 4.

The forget gate $f_{t}$ uses a sigmoid function to determine which information in the unit (Equation (7)) state will be forgotten or remembered.

(7) $$f_{t}=\sigma (W_{f}\times [h_{t-1},x_{t}]+b_{f})$$

In Equation (7), $f_{t}$ represents the output of the forget gate; σ is the sigmoid function; $W_{f}$ is the weight matrix of the forget gate; $h_{t-1}$ represents the final output of the cell state at the previous moment; $x_{t}$ is the input at the current moment; $b_{f}$ is the bias term of the forget gate.

The input gate $i_{t}$ is the second component, which determines which new information will be added to the cell state (Equation (8)).

(8) $$i_{t}=\sigma (W_{i}\times [h_{t-1},x_{t}]+b_{i})$$

(9) $${\tilde{C}}_{t}=\tanh(W_{C}\times [h_{t-1},x_{t}]+b_{C})$$

In Equations (8) and (9), $i_{t}$ is the output of the input gate; ${\tilde{C}}_{t}$ represents the backup storage unit at the current time; *tanh* s the hyperbolic tangent activation function; $W_{i}$ and $W_{C}$ the weight matrices of the output gate and backup storage unit, respectively; $b_{i}$ and $b_{C}$ are the bias terms of the output gate and backup storage unit.

The unit state $C_{t}$ is a stage in the memory unit storage process that uses the output values of the forgetting gate and the input gate (Equation (10)).

(10) $$C_{t}=f_{t}⊗C_{t-1}+i_{t}⊗{\tilde{C}}_{t}$$

In Equation (10), $C_{t}$ and $C_{t-1}$ represent the storage unit states at the current time *t* and time *t* − 1, respectively; ⊗ represents element-wise multiplication.

The output gate $o_{t}$ determines the output information based on the unit status (Equation (11)).

(11) $$o_{t}=\sigma (W_{o}\times [h_{t-1},x_{t}]+b_{o})$$

(12) $$h_{t}=o_{t}⊗\tanh(C_{t})$$

In Equation (11), $o_{t}$ is the output of the output gate; $h_{t}$ represents the hidden state at the current moment; $W_{o}$ is the weight matrix of the output gate; $b_{o}$ is the bias term of the output gate.

#### 3.2.2. PI-LSTM

**Figure 5:**
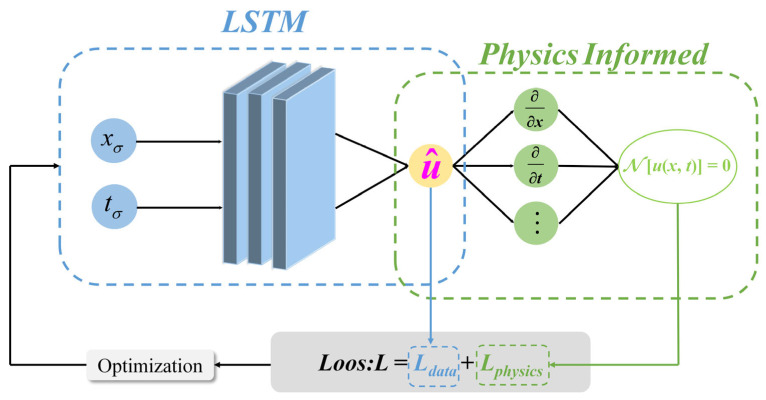
PI-LSTM network structure.

LSTM proves highly efficient when handling time series data, yet it relies heavily on the data. Shubhendu Kumar Singh et al. [42] propose a method called PI-LSTM, which combines LSTM with physical information, integrating data-driven approaches with physical information patterns to enable the model to adhere to physical law constraints in a data-driven manner. The structure of PI-LSTM is shown in Figure 5.

In the PI-LSTM architecture, physical constraints are embedded into the loss function of the neural network in the form of differential equations, transforming the traditional solution process into a differentiable optimization problem. This method achieves implicit identification of system parameters and explicit constraints on dynamic evolution by minimizing the joint loss of physical residuals and data errors. The loss function is expressed as in Equation (13):

(13) $$L=L_{data}+L_{physics}$$

where *L* is the total loss, *L_data_* is the data-driven loss, and *L_physics_* is the physics-information-driven loss. Based on the principle in partial differential Equation (14), the data-driven loss and physics-information-driven loss can be expressed as Equations (15) and (16).

(14) N[u(x,t)]=0

(15) $$L_{data}=\frac{1}{N}\sum_{i=1}^{N}{\left\|u_{p}(x_{i},t_{i})-u_{r}(x_{i},t_{i})\right\|}^{2}$$

(16) $$L_{physics}=\frac{1}{N}\sum_{i=1}^{N}{\left\|N\left[u_{p}(x_{i},t_{i})\right]\right\|}^{2}$$

In the equation, u(x,t) is the state variable describing the system, x is the spatial position, t is time, N is the physical operator, $u_{p}$ is the predicted value, and $u_{r}$ is the actual value.

The PI-LSTM model grasps the dynamics of input time series via the LSTM network and forecasts dependencies within the temporal dimension to minimize data-driven loss. At the same time, during model training, the physical constraints in the partial differential Equation (14) are enforced at configuration points, causing the physics-driven loss to simultaneously approach a minimum value. In this way, PI-LSTM combines the complementary advantages of time series modeling and physical constraints, making the model both robust and interpretable.

### 3.3. Real-Time Carbon Content Prediction Model for the Reblowing Stage of a Converter

#### 3.3.1. Architectural Design of PI-LSTM

**Figure 6:**
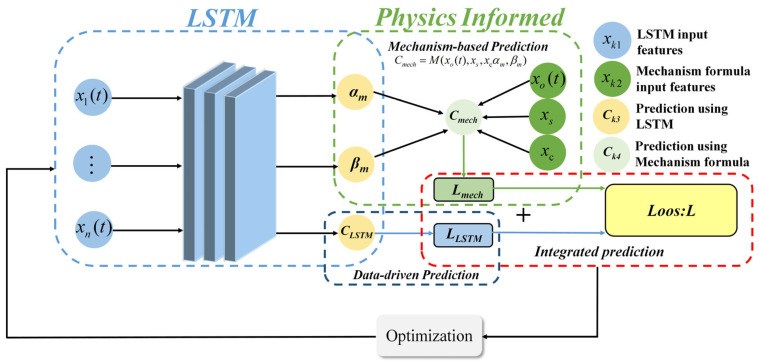
PI-LSTM for real-time prediction of carbon content during the reblowing stage of BOF.

In many current PI-LSTM applications, the parameters in the physical information equation are either measurable or empirical constants, and the results can be directly integrated into the LSTM for constraint purposes. However, in the converter decarburization mechanism Formula (6), *α_m_* and *β_m_* are critical unmeasurable parameters that vary with real-time smelting conditions. Therefore, based on the existing PI-LSTM framework, a network structure for PI-LSTM suitable for real-time prediction of carbon content during the converter’s reblowing stage was designed, as shown in Figure 6.

The PI-LSTM model consists of three components: mechanism-based prediction, data-driven prediction, and integrated prediction. The LSTM input features include metallurgical time-series data and single-value data. The time-series data includes oxygen lance height, oxygen supply flow rate, bottom blowing gas flow rate, and carbon content (actual carbon content during training and predicted carbon content during testing) during the reblowing stage; single-value data includes TSC carbon content and molten steel weight. TSC carbon content serves as the baseline for initial carbon content during the reblowing stage. At the same time, molten steel weight reflects the initial material conditions of the heat, collectively characterizing the differences in initial states across different furnace heats. The input features for the mechanistic formula are real-time cumulative oxygen consumption from time-series data, as well as single-value data, including TSC carbon content and molten steel weight.

In the data-driven section, a dual-hidden-layer LSTM network is designed to extract temporal features and perform dual-branch prediction using a sliding window prediction method, i.e., predicting based on the most recent fixed-length continuous time series data. The LSTM’s hidden state is projected via a fully connected layer for the direct prediction of the carbon content *C_LSTM_* in the next time step. The unobservable key parameters *α_m_* and *β_m_* in the decarbonization mechanism formula are predicted through an independent fully connected branch and projected onto an a priori knowledge interval to guarantee the parameter range and speed up convergence. In the mechanism-based part, the parameters *α_m_* and *β_m_* predicted by the data-driven branch, along with real-time cumulative oxygen consumption, TSC carbon content, and molten steel weight, are substituted into the converter decarbonization mechanism in Equation (6) to calculate the carbon content *C_mech_* under mechanism constraints.

Finally, in the integrated prediction section, the dual-branch outputs are fused through a collaborative constraint strategy, and the data-driven prediction results and the mechanism-based prediction results are weighted and fused to obtain the final integrated prediction results.

The joint loss function of the model is designed as the sum of the loss of the data-driven part and the loss of the mechanism-based part. The model simultaneously fits the data patterns and mechanism patterns, as shown in Equation (17). The final integrated prediction result calculation formula is shown as follows:

(17) $$L=L_{LSTM}+L_{mech}$$

(18) $$C_{pre}=\lambda C_{LSTM}+(1-\lambda )C_{mech}$$

Among these, *L* represents the joint loss, *L_LSTM_* denotes the loss for data-driven prediction, similar to the data-driven loss *L_data_* in the standard PI-LSTM. *L_mech_* is the loss based on mechanism-constraint prediction, analogous to the physics-information-driven loss *L_physics_* in the standard PI-LSTM. *C_pre_* is the final predicted carbon content value, *λ* is the weighting coefficient, *C_LSTM_* denotes the data-driven predicted carbon content value, and *C_mech_* represents the mechanism-based predicted carbon content value. When *C_LSTM_* approaches the actual value, *L_LSTM_* decreases. In response to changes in metallurgical conditions such as gun position and gas flow, LSTM optimizes the unmeasurable parameters of the mechanistic formula in real time, thereby reducing the error between *C_mech_* and the actual value, leading to a decrease in Lmech. The final predicted carbon content *C_pre_* is designed as the weighted fusion result of data-driven and mechanistic constraint-based predictions.

Given the impact of sensor noise and process fluctuations on converter smelting data, unlike the standard PI-LSTM, which uses mean square error to calculate loss, the strong robustness of mean absolute error (MAE) can improve the reliability of model control [19]. The calculation formula is as follows:

(19) $$L_{LSTM}=\frac{1}{N}\sum_{i=1}^{N}\left|C_{LSTMi}-C_{i}\right|$$

(20) $$L_{mech}=\frac{1}{N}\sum_{i=1}^{N}\left|C_{mechi}-C_{i}\right|$$

#### 3.3.2. Model Implementation Process

**Figure 7:**
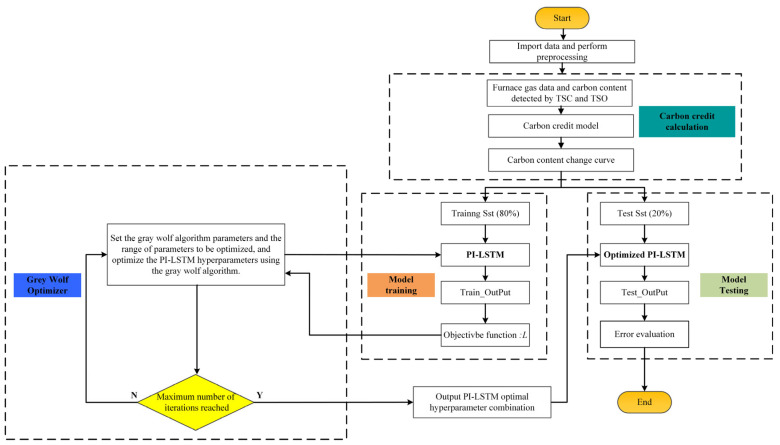
Flowchart of real-time carbon content prediction model based on PI-LSTM for the reblowing stage of the converter.

Hyperparameter optimization is crucial for LSTM, as it significantly affects the model convergence speed and ultimate performance. Compared to grid search and random search, heuristic algorithms offer superior efficiency and global optimization capabilities. The gray wolf optimization (GWO) algorithm is a heuristic algorithm based on swarm intelligence proposed by Mirjalili et al. [43]. It mimics the hunting behavior of gray wolves in nature, transforming complex optimization problems into the distance relationship between gray wolves and prey. Through multiple iterations, the distance gradually decreases, ultimately yielding the optimal solution [44]. The execution flowchart of the real-time carbon content forecasting model for the converter reblowing stage grounded in PI-LSTM is illustrated in Figure 7.

The data preprocessing section includes extracting single-valued data such as molten steel weight and TSC content, as well as time-series data such as cumulative oxygen consumption, oxygen flow rate, bottom-blown gas flow rate, oxygen lance height, and carbon content. The data is normalized, and the time-series data is processed using a sliding window.

In the carbon credit calculation section, the carbon content change curve is calculated using the carbon credit model based on aligned converter flue gas data and the carbon content values of TSC and TSO, which are used as the actual carbon content values.

The data is split into a training set and test set at an 8:2 proportion. In the model training section, a dual-branch collaborative optimization mechanism combining data-driven and mechanism-based calculations is employed. The input includes the oxygen lance height, oxygen flow rate, bottom-blown gas flow rate, and carbon content calculated from the carbon integration over a fixed number of consecutive time points, as well as static data such as TSC carbon content and molten steel volume. Through dual-branch parallel prediction using PI-LSTM, the data-driven branch outputs direct predictions of carbon content and mechanism parameters *α_m_* and *β_m_* via LSTM. The mechanism prediction part combines molten steel weight and cumulative oxygen consumption with unmeasurable parameters to calculate carbon content based on mechanism prediction. The optimization objective is a joint loss function (simultaneously minimizing the error between data-driven prediction and actual values, and the error between mechanism calculation prediction and actual values). Through iterative updates of model parameters, the prediction results of both branches are synchronously approximated to the actual values. Additionally, the GWO algorithm is introduced to search for LSTM hyperparameters (such as learning rate and number of hidden layer neurons), using the joint loss as the fitness function. By comparing the performance of the model under different parameter combinations, the optimal hyperparameter configuration is determined, ultimately achieving the synergistic optimization of data-driven capabilities and mechanistic constraints.

In the model testing section, an autoregressive weighted fusion prediction mechanism is employed. Starting from the TSC time point, the following time-series parameters are input into the trained and optimized PI-LSTM model: oxygen lance height, oxygen flow rate, bottom-blown gas flow rate, cumulative oxygen consumption, and carbon content at the corresponding time points (the carbon content at the TSC time point is the actual carbon content at the TSC time point, while the carbon content at other time points is the weighted fusion-predicted carbon content). Additionally, static features such as TSC carbon content and molten steel weight are also input into the model. The model simultaneously outputs the data-driven predicted value for the next time point and the parameters required for mechanism-based calculations. The final predicted value for the current time point is then obtained through a weighted fusion strategy. During iterative prediction, the new predicted value is appended to the end of the input sequence, and the earliest time point data in the sequence is removed to form a new input sequence (maintaining consistent input length); Combining the real-time updated temporal operational parameters and the constant static features, the above prediction process is repeated iteratively until the TSO time point, ultimately forming a complete carbon content change curve; The accuracy of the model is evaluated by comparing this predicted curve with the actual curve calculated by the carbon integration model.

## 4. Model Results and Analysis

### 4.1. Data Preprocessing

This study employs practical production data gathered from a steel mill’s 300t low-carbon, low-phosphorus steel grade SDC as the research subject. To mitigate the impact of anomalous measurements on model performance, we cleansed the data by identifying and excluding outliers based on the Interquartile Range (IQR) criterion. This method is preferred for its robustness against non-normal data distributions. After sorting the data, we computed Q1 (25th percentile) and Q3 (75th percentile). The IQR (IQR = Q3 − Q1), a measure of statistical dispersion, was then used to establish a lower bound (Q1 − 1.5 × IQR) and an upper bound (Q3 + 1.5 × IQR). Observations outside this interval were classified as outliers and discarded. Applying this filter produced a refined dataset of 1207 high-quality heat records. The data was randomly partitioned, with 80% allocated as the training set and 20% as the test set.

The statistical results of the single-valued and reblowing stage time-series data from the converter blowing process are shown in Table 1. The processed data for 1207 heats all pertain to the low-carbon, low-phosphorus steel grade SDC. The initial carbon content (*C_TSC_*) ranged from 0.064% to 0.554% (mean 0.244%, standard deviation 0.1068%), while the final carbon content (*C_TSO_*) ranged from 0.025% to 0.066% (mean 0.042%, standard deviation 0.00782%). The composition range covered the typical smelting requirements for this steel grade with no extreme deviations. Key process parameters (oxygen flow rate 28,164–64,228 Nm^3^·h^−1^; oxygen lance height 1498–5580 mm; bottom-blown gas flow rate 424–2868 Nm^3^·h^−1^) all fall within the standard operating range for 300-ton converters. The smelting cycle during the re-blowing phase lasted 1–3 min, consistent with industrial production practices.

Among them, *C_TSC_* and *C_TSO_* are the carbon content detected at TSC time and TSO time, respectively.

**Figure 8:**
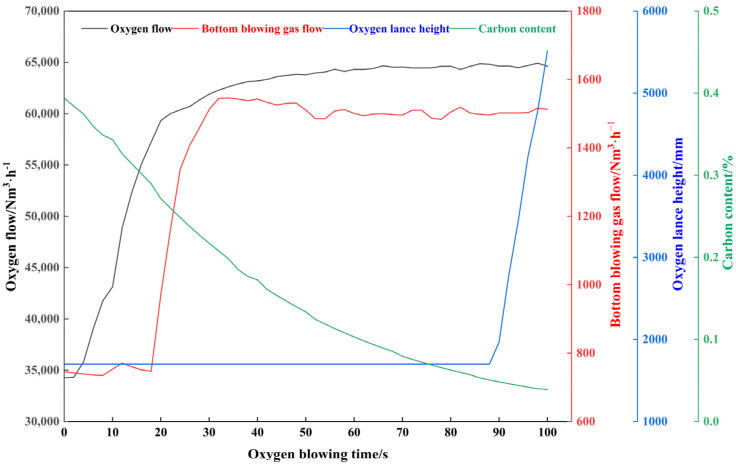
Time-series data during the reblowing stage of a certain heat.

To address the issue of smoke data lag, the carbon content calculated from smoke data will be aligned with non-delayed time-series data, such as oxygen flow rate. The on-site data delay is 70 s. The time-series data for the reblowing stage of a certain heat after alignment is shown in Figure 8:

Min-max normalization is employed to guarantee that data is scaled to a consistent range of [0, 1] in modeling, preventing gradient instability resulting from varying dimensions of process parameters, speeding up model convergence, and enhancing training efficiency and prediction stability.

(21) $$Z^{\prime }=\frac{Z-Z_{\min}}{Z_{\max}-Z_{\min}}$$

In Equation (21), $Z^{\prime }$ is the standardized data, Z is the original data, $Z_{\max}$ is the maximum value of the original data, and $Z_{\min}$ is the minimum value of the original data.

**Figure 9:**
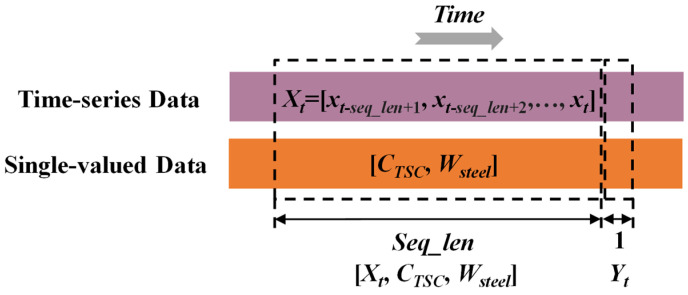
Sliding window processing data.

In real-time carbon content prediction scenarios, the temporal dependency of time-series data is critical. To effectively utilize this characteristic, a sliding window mechanism is employed to structurally process continuous data [45], thereby generating data pairs consisting of input sequences and output labels. The processing workflow is illustrated in Figure 9. For time-series features, the time-series feature observations from the most recent fixed number of consecutive time points in each data pair form the input sequence *X_t_* = [*x_t_*_-*seq_len*+1_, *x_t_*_-*seq_len*+2_*, …, x_t_*], with the corresponding output label *Y_t_* being the carbon content value at the next time point. The input sequence length *seq_len* is determined through hyperparameter optimization. Additionally, single-valued data such as TSC carbon content and molten steel volume are concatenated with the temporal input of each data pair to form the complete input vector [*X_t_*, *C_TSC_*, *W_steel_*]. The large number of overlapping samples generated in this manner can expand the training set, enhancing the model’s generalization ability. During the prediction phase, the window is dynamically updated to track changes in carbon content in real time.

### 4.2. Model Evaluation Indicators

In the evaluation of model prediction performance, the root mean square error (RMSE), mean absolute error (MAE), and hit rate (HR) of endpoint carbon content are used as key indicators. RMSE quantifies the overall error level between the predicted values and the actual values by calculating the square root of the mean of the squared differences between the predicted values and the actual values. The magnitude of the RMSE directly reflects the overall deviation of the prediction results, with smaller values indicating higher prediction accuracy; MAE measures the average size of prediction errors by calculating the average of the absolute values of the deviations between predicted and actual values. MAE is less affected by extreme errors and reflects the central tendency of prediction errors; HR is used to assess the accuracy of the model’s prediction of the final carbon content in smelting. It is defined as the proportion of furnace runs where the deviation between the predicted and actual endpoint carbon content falls within a predefined acceptable range, as shown in the formula below (Equations (22)–(24)):

(22) $$RMSE=\sqrt{\frac{1}{n}\sum_{i=1}^{n}{\left(y_{i}-\overset{\wedge }{y_{i}}\right)}^{2}}$$

(23) $$MAE=\frac{1}{n}\sum_{i=1}^{n}\left|y_{i}-\overset{\wedge }{y_{i}}\right|$$

(24) $$HR=\frac{\eta }{n}\times 100\%$$

In Equations (22)–(24), $y_{i}$ represents the actual carbon content value, $\overset{\wedge }{y_{i}}$ represents the model-predicted carbon content value, η represents the number of furnaces where the absolute value of the difference between the predicted endpoint carbon content and the TSO carbon content does not exceed ±0.005%, ±0.01%, or ±0.015%; and *n* represents the total number of heats participating in the model prediction.

### 4.3. PI-LSTM Model Hyperparameter Optimization

**Figure 10:**
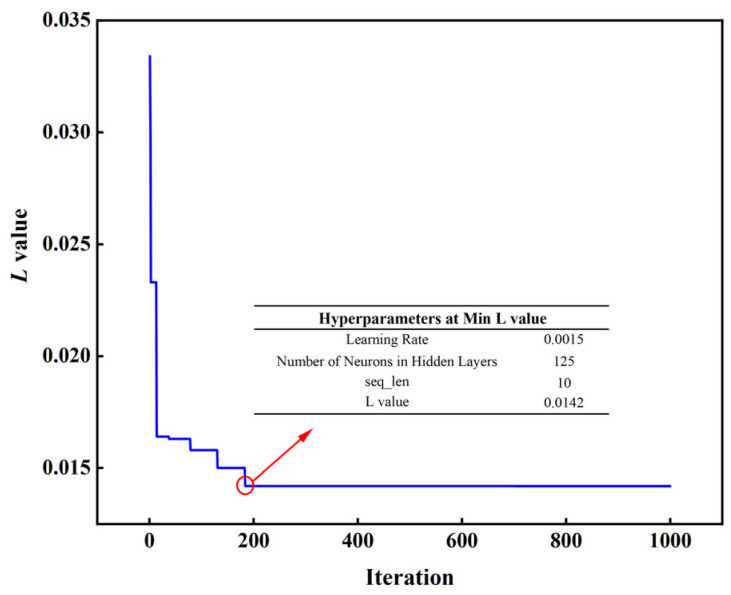
GWO iterative optimization process.

To enhance the convergence speed, predictive accuracy, and generalization performance of the PI-LSTM model, GWO is employed to tune the hyperparameters of LSTM, including learning rate, hidden layer neuron count, and sequence length (seq_len). The learning rate regulates the step size of weight adjustments in model training. If excessively large, it might lead to training oscillations or divergence; if overly small, convergence will be sluggish. The setting range is [0.0001, 0.1]. The number of neurons in the hidden layer determines the capacity and complexity of the LSTM network. Too few neurons may result in underfitting, unable to capture complex temporal patterns; too many may lead to overfitting. Considering model capacity and computational efficiency, the setting range is [10, 500]. The input sequence length in the sliding window mechanism affects the model’s ability to capture time dependencies. Too small a length may ignore long-term dependencies, while too large a length increases computational burden and may introduce noise. Based on the data sampling frequency (0.5 Hz, i.e., one sample every 2 s) and the duration of the reblowing stage of the converter furnace (1~3 min), the setting range is [1, 15]. The configuration of the GWO algorithm is as follows: the gray wolf population size is set to 30, the maximum iteration count is 1000, and the fitness function adopts the joint loss *L* specified in Equation (17). The training set data is fed into the model, with the iterative process illustrated in Figure 10.

GWO converged to the global optimal solution after only 185 iterations, with the joint loss value *L* stabilizing at 0.0142, fully demonstrating its efficiency in hyperparameter optimization, particularly in complex nonlinear scenarios such as carbon content prediction during the reblowing stage of a converter. The final optimal parameter configuration determined was a learning rate of 0.0015, 125 hidden layer neurons, and an input sequence length of 10: The learning rate balances the model’s sensitivity to dynamic changes in carbon content with convergence stability; the number of neurons enhances the ability to extract multi-scale features (such as short-term operational fluctuations and long-term decarbonization trends), avoiding overfitting while improving the expression of temporal features; the sequence length of 10 corresponds to a 20 s historical window, aligning with the delay characteristics of decarbonization reactions, ensuring effective capture of lag effects. Overall, GWO optimization significantly improves the predictive performance of the PI-LSTM model.

The optimized model’s joint loss value *L* is 0.0142, where the loss *L_LSTM_* for data-driven prediction is 0.0057, and the loss *L_mech_* for mechanism-based prediction is 0.0085. To determine the weighting coefficient *λ* for result fusion in Equation (18), the calculation is performed using Equation (25). The final adjustment of *λ* is 0.6, meaning that the data-driven branch prediction accounts for 60% of the weight, and the mechanism-based prediction accounts for 40% of the weight. This allocation reflects the dominance of the data-driven branch due to its lower loss, fully leveraging its ability to capture the nonlinear relationships of temporal operational parameters; it also retains the necessary proportion of the mechanistic model, ensuring that the prediction results do not deviate from the decarbonization kinetic laws, even though its loss is relatively larger. The 40% weight ensures that the prediction results do not deviate from the decarbonization kinetic laws, avoiding potential physical and logical deviations that may arise from data-driven predictions, ultimately achieving a dynamic balance between data features and mechanistic constraints.

(25) $$\lambda =\frac{L_{mech}}{L_{LSTM}+L_{mech}}$$

### 4.4. Model Results Analysis

#### 4.4.1. Analysis of Prediction Results

**Figure 11:**
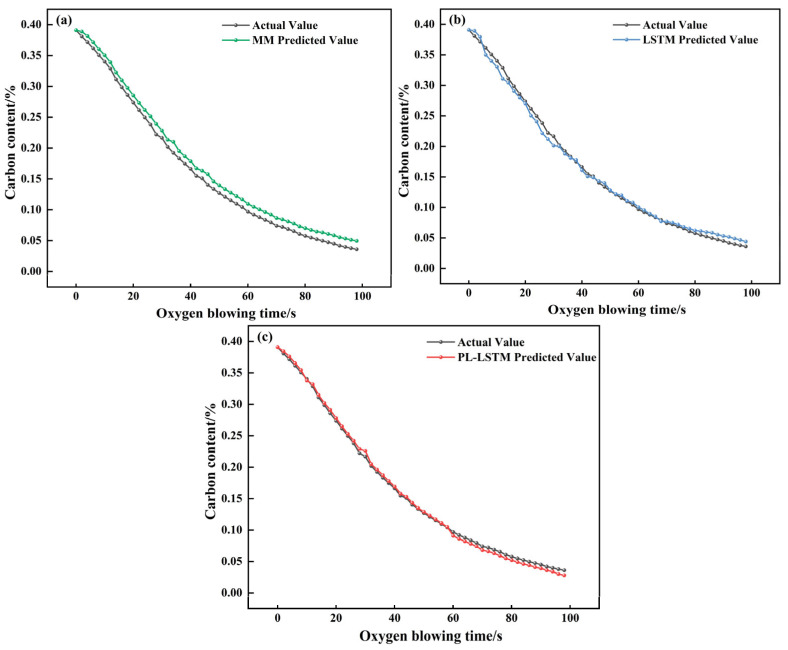
Comparison of real-time prediction results and actual results for the typical heat using (**a**) MM, (**b**) LSTM, and (**c**) PI-LSTM.

**Figure 12:**
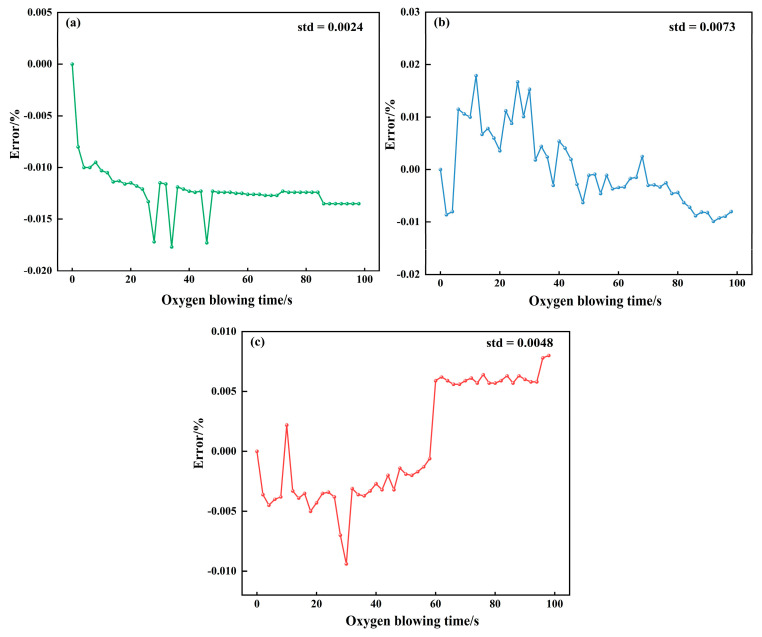
Real-time prediction error of carbon content for the typical heat using (**a**) MM, (**b**) LSTM, and (**c**) PI-LSTM.

**Figure 13:**
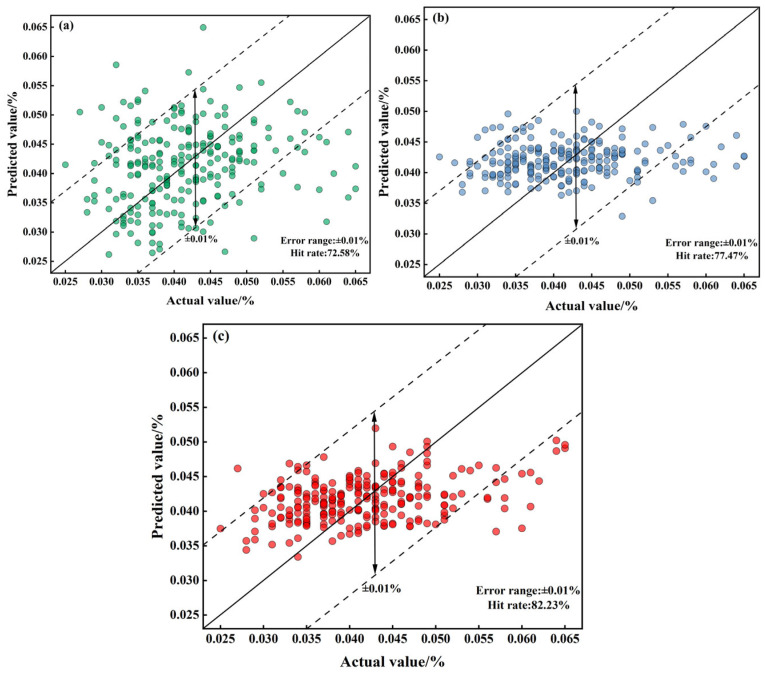
Scatter plot of actual and predicted endpoint carbon content values using (**a**) MM, (**b**) LSTM, and (**c**) PI-LSTM.

The predictive performance of the constructed PI-LSTM model was verified by comparing it with a mechanistic model (MM) and a purely data-driven LSTM model (to ensure fairness, the unobservable parameters of MM and the hyperparameters of LSTM were optimized based on the training set using the GWO algorithm). Additionally, results from the self-attention-based convolutional parallel network (SabCP) carbon content prediction model from reference [22], the CBR-LSTM carbon content prediction model from reference [12], and the PINN carbon content prediction model from reference [19] were introduced for comparative analysis with the model proposed in this paper. The results are shown in Table 2, Figure 11, Figure 12 and Figure 13.

The mechanism-based model realizes prediction by virtue of the converter’s decarbonization kinetic formula, achieving an MAE of 0.0172 and an RMSE of 0.0207. Its hit rates within the error margins of ±0.005%, ±0.01%, and ±0.015% stand at 38.84%, 72.98%, and 89.53%, respectively. From the typical furnace prediction curve (Figure 11a), it can be seen that although the model optimizes the key parameters in the decarbonization process using GWO, it is difficult to adapt to the dynamic changes in real-time smelting conditions, resulting in significant numerical deviations between the predicted curve and the actual value. It cannot capture the short-term fluctuations in carbon content caused by operations such as oxygen lance adjustment and bottom blowing flow rate fluctuations, and has a significant deviation from the actual value. Considering the typical heat error curve (Figure 12a), the standard deviation of the error in the mechanism model is only 0.0024. Although the fluctuation range is small, the error sequence exhibits a significant systematic offset (errors concentrated between −0.015% and −0.020%) with no dynamic correction trend. This fundamentally stems from the fixed decarburization formula’s inability to match the real-time reaction state of the molten pool. The scatter plot of endpoint carbon content (Figure 13a) shows that the predicted results are relatively scattered, with some points exceeding the ±0.01% error range. This reflects that although the mechanism model can follow the overall decarbonization law, it is difficult to adapt to the differences in smelting conditions of different furnaces, and its performance is limited in high-precision control scenarios.

The LSTM model captures temporal features through a purely data-driven approach, with an MAE of 0.0098 and an RMSE of 0.0134. The hit rates within the error ranges of ±0.005%, ±0.01%, and ±0.015% are 45.03%, 77.47%, and 92.14%, respectively. The pure LSTM model is more accurate than the mechanistic model but suffers from a critical flaw. It lacks knowledge of physical laws. Since it is not constrained by the principle that carbon content must decrease monotonically during blowing, its predictions can show unphysical fluctuations, such as temporary increases ((Figure 11b). Furthermore, as shown in Figure 12b, its prediction error is unstable and can change abruptly. While most of its endpoint predictions are accurate (Figure 13b), it produces significant errors in scenarios rarely seen in the training data (e.g., very low carbon content), a classic sign of overfitting. This demonstrates that purely data-driven models are highly dependent on data quality and have limited generalization capability.

The PI-LSTM model integrates the temporal modeling capability of LSTM with decarbonization mechanism constraints, achieving the best performance with an MAE of 0.0077 and an RMSE of 0.0112. The hit rates within the error ranges of ±0.005%, ±0.01%, and ±0.015% are 53.71%, 82.23%, and 95.45%, respectively. The PI-LSTM model demonstrates superior performance, as its predictions almost perfectly match the actual measurements (Figure 11c). This is achieved by its dual-branch design: one branch learns from real-time data to capture process dynamics, while the other enforces physical laws to ensure the predictions follow a realistic exponential decay, consistent with the three-stage decarbonization theory. The model’s exceptional stability is evident in its error profile (Figure 12c), which has a very low standard deviation (0.0048) and fluctuates randomly within a narrow band around zero, showing no systematic bias. Finally, the scatter plot (Figure 13c) confirms its high precision, with nearly all endpoint predictions falling within the ±0.01% error margin. This shows that the model successfully balances general physical principles with the specifics of each heat, delivering accurate, reliable, and physically plausible predictions for real-time control.

Furthermore, comparisons with other literature methods reveal that: Compared to the SabCP [22] data-driven soft measurement model, the PI-LSTM model achieves approximately 6.73% improvement in hit rate at ±0.01% (from 75.50% to 82.23%) and approximately 8.55% improvement at ±0.015% (from 86.9% to 95.45%). This improvement stems from the integration of mechanism-based constraints, which mitigates the physical inconsistencies inherent in data-driven predictions. Compared to PINN [12] (Physical Information Neural Network Hybrid Model), PI-LSTM achieves a ±0.005% accuracy improvement of approximately 3.49% (from 50.22% to 53.71%), with a low MAE of 0.0077. This is attributed to the LSTM gating mechanism’s enhanced ability to accurately capture the impact of dynamic temporal features—such as oxygen lance position adjustments during the re-blowing phase—on carbon content. Compared to CBR-LSTM [19] (Case-Based Reasoning Hybrid Model), PI-LSTM achieves approximately 29.23% improvement in ±0.01% accuracy (from 53.00% to 82.23%) and approximately 23.45% improvement in ±0.015% accuracy (from 72.00% to 95.45%). The model’s dual-branch architecture enables direct optimization of unobservable parameters in the mechanistic model without relying on case matching, which exhibits poor adaptability to new smelting conditions.

**Figure 14:**
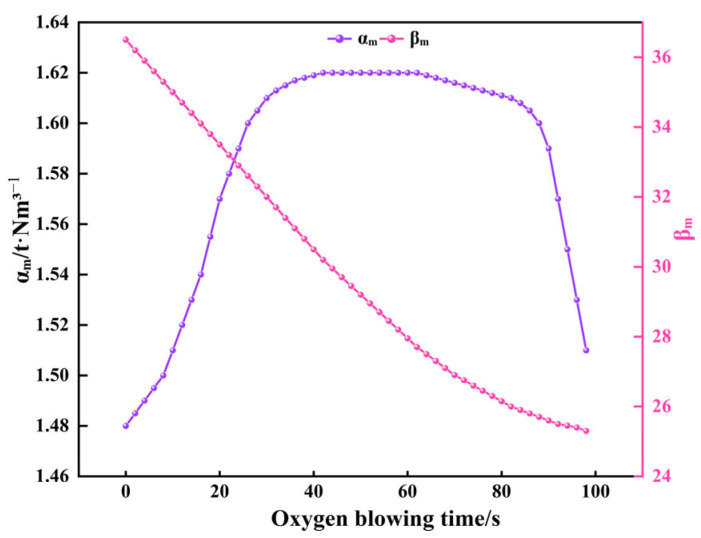
Real-time variation in mechanism parameters for typical furnace cycles using PI-LSTM.

To validate the validity of the mechanism parameter prediction results in the PI-LSTM model, the real-time changes in key parameters for a typical furnace operation are presented, as shown in Figure 14.

Combining Figure 8 and Figure 14 reveals that the real-time predicted values of αₘ and βₘ in a typical furnace cycle exhibit dynamic changes highly consistent with the process operations during the secondary blowing stage and the decarbonization kinetics. αₘ remained within the range [1.48, 1.62] throughout the process, consistently falling within the theoretical range αₘ ∈ [1, 2] reported in reference [37]. Its trend precisely responded to operational adjustments: it rose steadily from 0 to 20 s as oxygen supply intensity increased, reflecting enhanced oxygen utilization efficiency; reaching a peak and maintaining high levels during the stable operation period from 20 to 80 s; then steadily decreasing from 80 to 100 s as the oxygen lance was raised and oxygen supply weakened, aligning with the requirements of the diffusion-controlled phase in the late blowing stage. βₘ ranged between [25.2, 36.5], fully consistent with the expected range of βₘ ∈ [20, 40] reported in reference [19]. It exhibited a monotonically decreasing trend with decreasing carbon content, perfectly aligning with the kinetic characteristic where decarburization reaction rates slow down due to carbon mass transfer limitations in the final stage. No abnormal fluctuations contrary to process logic were observed, validating the rationality of the model predictions.

#### 4.4.2. Impact of Measurement Errors on the Model

In the carbon content prediction model for the second blowing stage of the converter, the carbon content at the TSC time and the molten steel weight are two irreplaceable key input parameters. Measurement errors in these parameters directly determine the accuracy of the model’s initial inputs and the reliability of subsequent predictions. Industrial TSC carbon content is measured by auxiliary guns, introducing systematic errors. Additionally, random errors may arise from variations in auxiliary gun insertion depth and molten pool inhomogeneity during actual smelting. This makes carbon content one of the single-valued parameters in model inputs that exhibit high sensitivity to prediction outcomes. Industrial molten steel weight is indirectly calculated from the charge ratio of pig iron and scrap steel, making it susceptible to errors due to weighing accuracy during charging—a parameter prone to inaccuracies within initial material conditions.

Industrial measurement errors must balance realism and gradient characteristics. A 5% error represents mild to moderate common industrial deviations, while a 10% error signifies moderate to extreme industrial deviations. This gradient clearly defines the model’s robustness boundaries. Errors typically follow a normal distribution to ensure randomness, as illustrated by Equations (26) and (27).

(26) $$C_{TSC,noisy}=C_{TSC}\times (1+\varepsilon )$$

(27) $$W_{steel,noisy}=W_{steel}\times (1+\varepsilon )$$

In Equations (26) and (27), $C_{TSC}$ denotes the carbon content at the original TSC time, $W_{steel}$ denotes the original molten steel weight, ε denotes a random variable following a normal distribution, $\varepsilon \sim N(0,\sigma^{2})$, or σ=0.05 (5% noise) or σ=0.10 (10% noise), $C_{TSC,noisy}$ denotes the carbon content at the TSC time after noise addition, and $W_{steel,noisy}$ denotes the molten steel weight after noise addition.

To validate the fundamental stability of the PI-LSTM model under noisy conditions, the study introduced various types and intensities of noise into the model inputs and conducted retests. The final results are presented in Table 3.

Whether adding 5% noise to the carbon content at the TSC moment or to the molten steel weight, the accuracy decline is limited, demonstrating the model’s reliability under normal industrial tolerances. When 10% noise was added, TSC carbon content caused MAE to increase from 0.0077 to 0.0092 (19.5% increase), and the ±0.01% hit rate decreased from 82.23% to 75.67% (8.0% decrease). In contrast, adding 10% noise to molten steel weight only increased MAE to 0.0088 (14.3% increase) while reducing ±0.01% hit rate to 77.85% (5.3% decrease). TSC represents the initial carbon content, where errors propagate continuously through the carbon integration model and mechanism formula. Meanwhile, molten steel weight corrects partial deviations in real-time via the model’s mechanism branch (based on the decarburization formula) by accumulating oxygen consumption, thereby mitigating error impacts.

When 5% noise was simultaneously added to both TSC-time carbon content and molten steel weight, the ±0.01% accuracy rate reached 78.14%, with errors remaining within acceptable limits. When 10% noise was added, MAE is 0.102, still significantly lower than the mechanism model’s MAE is 0.0172. The ±0.015% accuracy rate of 90.27% continues to meet industrial requirements.

The strong robustness of PI-LSTM against measurement errors stems from two aspects: firstly, MAE insensitivity to extreme errors prevents significant accuracy degradation due to input errors in individual heats; secondly, even when model inputs contain errors, the mechanism branch corrects deviations in the data-driven branch based on physical laws (such as the monotonic decrease of carbon content during the reblowing phase and decarburization rates conforming to the three-stage theory) ensuring predictions remain consistent with metallurgical principles.

#### 4.4.3. Model Generalization Analysis

During converter steelmaking, process parameters and composition control targets vary across different steel grades. A key criterion for assessing a model’s industrial applicability is its ability to maintain stable prediction accuracy when applied to the same equipment but different steel grades. To validate the generalization capability of the PI-LSTM model, weathering steel SPA-H whose process characteristics differ from the original study subject (low-carbon, low-phosphorus steel SDC) was selected as the test subject. The data preprocessing workflow was identical to that for SDC steel (including IQR outlier removal, Min-Max normalization, sliding window time series reconstruction, and 8:2 random division of training and test sets) to ensure testing fairness. The processed dataset comprised 1012 heats. Statistical results for key input parameters of SPA-H steel are shown in Table 4.

The smelting process parameters for SPA-H steel grade (such as oxygen flow rate and oxygen lance height) differ from those of SDC steel grade, primarily reflected in a higher maximum oxygen supply flow rate (to accommodate the decarburization requirements of weather-resistant steel) and a slightly higher initial carbon content. These differences simulate the actual production scenario of different steel grades processed on the same equipment in industrial settings, effectively testing the model’s adaptability to variations in parameter distributions.

For the 1012 heats data of SPA-H steel, the PI-LSTM model structure (dual-branch network), hyperparameter optimization method (GWO), and loss function were maintained consistent with those for SDC steel. After independent training, the optimized SPA-H-specific model was applied to its test set. The prediction results compared with those from the SDC steel model are shown in Table 5.

As shown in Table 5, after generalizing the PI-LSTM model to the SPA-H steel grade, the model achieved an MAE of 0.0089 and an RMSE of 0.0125 on external data, with a ±0.01% error hit rate of 79.50%. Although its performance is slightly lower than that on the original data, it still meets industrial accuracy requirements. This effectively reduces dependence on specific steel grade data and demonstrates potential for application across multiple steel grades within the same equipment.

#### 4.4.4. Scope of Model Applicability

The application scope and validation prerequisites of the PI-LSTM model developed in this study are defined based on the following conditions:

Equipment Boundary: The model is applicable to 300 t nominal capacity converters. If applied to small converters below 120 t or large converters above 500 t, model hyperparameters must be re-optimized using GWO.

Steel Grade Boundary: The model is suitable for low-carbon steels, including low-carbon low-phosphorus steels (SDC type) and weather-resistant steels (SPA-H type).

Data Boundary: The application requires the availability of both single-value process data and time-series data, supported by flue gas analysis system data.

Model Validation and Comparison: Validation and comparative analysis with other models (e.g., MM, LSTM) must be conducted under consistent equipment specifications (300t converter) and similar steel grade compositions (low-carbon steel). This ensures the avoidance of accuracy comparison distortions caused by differences in equipment or steel grade.

## 5. Conclusions

This paper focuses on the challenge of the real-time forecasting of carbon content in a converter’s reblowing phase. An innovative PI-LSTM model integrating data-driven and mechanism-constrained approaches has been developed to enable real-time forecasting of carbon content changes during the reblowing phase, providing a basis for dynamic adjustments in the steelmaking process. The main research conclusions are as follows:

(1) A dual-branch PI-LSTM structure was proposed. The data-driven branch used LSTM to capture the dynamic characteristics of sequential operational parameters (such as oxygen flow rate and oxygen lance height), simultaneously predicting carbon content and key parameters of the decarburization kinetics. The mechanism-based branch calculated carbon content under mechanism constraints using key parameters and decarburization formulas, achieving synergistic optimization between data fitting and mechanism constraints. This addressed the issues of insufficient physical consistency in pure data models and limited accuracy in mechanism-based models.
(2) The gray wolf algorithm was used to optimize the LSTM hyperparameters: a learning rate of 0.0015, a hidden layer neuron count of 125, and an input sequence length of 10. The joint loss function converged to 0.0142, balancing the data-driven loss (0.0057) and mechanism-constrained loss (0.0085), thereby improving model convergence speed and prediction stability.
(3) Tests on converter data showed that the MAE of PI-LSTM was 0.0077, a 55.2% reduction compared to the MAE of the mechanism model (0.0172) and a 21.4% reduction compared to the MAE of LSTM (0.0098). The hit rate for endpoint carbon content within a ±0.01% error range reached 82.23%, significantly outperforming the comparison models, and the prediction curve accurately tracked the dynamic changes in carbon content, consistent with the physical laws of the three-stage decarburization theory.
(4) The PI-LSTM model demonstrates the strong robustness and cross-scenario generalization capability required in industrial applications. On one hand, when faced with common measurement errors in the field, the model maintains a hit rate of over 90% within a ±0.015% error margin, effectively resisting disturbances such as sublance detection deviations and charging weight inaccuracies. On the other hand, when generalizing from low-carbon low-phosphorus steel SDC to weather-resistant steel SPA-H, which has significantly different process characteristics, the model MAE only increases from 0.0077 to 0.0089, while the ±0.01% hit rate remains at 79.50%. This capability enables the model to adapt to complex industrial conditions, including different steel grades and sensor noise.

The PI-LSTM model developed in this study demonstrates key advantages, including high real-time prediction accuracy, enhanced interpretability through the integration of physical mechanisms, and strong practical applicability in industrial settings. The model exhibits good robustness against measurement errors and variations in steel grades, providing reliable support for real-time closed-loop control.

However, the model also has certain limitations. Firstly, its training and validation are based on data from a specific 300-ton converter at a single steel plant and limited steel grades (SDC and SPA-H). Its applicability to larger converters and steel grades with higher carbon content requires further verification. Secondly, although the model reduces data dependency through mechanism-based constraints, it still requires sufficient high-quality historical data for training.

Future work will focus on collecting more diverse industrial data to evaluate the model’s generalization capability and exploring integration with sensor error estimation techniques to further enhance its stability in complex industrial environments.

## Figures and Tables

**Table 1:** Statistical results of converter smelting data.

Symbol	Data Type	Variables	Sampling Frequency	Min Value	Max Value	Mean	Standard Deviation
*X*_1_	singl-valued	molten steel weight/t	/	278	314	300.12	6.32
*X*_2_	singl-valued	*C_TSC_*/%	/	0.064	0.554	0.244	0.1068
*X*_3_	time-series	oxygen flow rate/Nm^3^·h^−1^	0.5/Hz	28,164	64,228	/	/
*X*_4_	time-series	oxygen lance height/mm	0.5/Hz	1498	5580	/	/
*X*_5_	time-series	bottom-blown gas flow rate/Nm^3^·h^−1^	0.5/Hz	424	2868	/	/
*X*_6_	time-series	cumulative oxygen consumption/Nm^3^	0.5/Hz	0	2534	/	/
*Y*_1_	singl-valued	*C_TSO_*/%	/	0.025	0.066	0.042	0.0078
*Y*_2_	time-series	Carbon content (carbon credit calculation)/%	/	0.026	0.544	/	/

**Table 2:** Carbon content prediction accuracy of different models.

Model Name	MAE	RMSE	HR/%		
			±0.005%	±0.01%	±0.015%
MM	0.0172	0.0207	38.84	72.98	89.53
LSTM	0.0098	0.0134	45.03	77.47	92.14
SabCP [22]	-	-	-	75.50	86.9
CBR-LSTM [12]	-	-	25.00	53.00	72.00
PINN [19]	-	-	50.22	81.37	93.52
PI-LSTM	0.0077	0.0112	53.71	82.23	95.45

**Table 3:** Measurement Error Analysis Results.

Error Type	MAE	RMSE	HR/%		
			±0.005%	±0.01%	±0.015%
No noise	0.0077	0.0112	53.71	82.23	95.45
*C_TSC_* + 5% noise	0.0083	0.0120	49.85	79.32	94.18
*C_TSC_* + 10% noise	0.0092	0.0135	45.21	75.67	92.03
*W_steel_* + 5% noise	0.0081	0.0118	51.06	80.59	94.72
*W_steel_* + 10% noise	0.0088	0.0128	47.93	77.85	93.15
*C_TSC_* + *W_steel_* + 5% noise	0.0087	0.0125	48.32	78.14	93.86
*C_TSC_* + *W_steel_* + 10% noise	0.0102	0.0147	42.57	73.29	90.27

**Table 4:** Statistical results for SPA-H steel grade.

Data Type	Variables	Sampling Frequency	Min Value	Max Value	Mean	Standard Deviation
singl-valued	molten steel weight/t	/	275	310	299.35	6.21
singl-valued	*C_TSC_*/%	/	0.059	0.550	0.255	0.1043
time-series	oxygen flow rate/Nm^3^·h^−1^	0.5/Hz	28,320	68,418	/	/
time-series	oxygen lance height/mm	0.5/Hz	1599	5578	/	/
time-series	bottom-blown gas flow rate/Nm^3^·h^−1^	0.5/Hz	444	2674	/	/
time-series	cumulative oxygen consumption/Nm^3^	0.5/Hz	0	2196	/	/
singl-valued	*C_TSO_*/%	/	0.026	0.067	0.043	0.0084
time-series	Carbon content (carbon credit calculation)/%	/	0.0267	0.535	/	/

**Table 5:** Comparison of model prediction results for SDC and SPA-H steel grades.

Steel Grade	MAE	RMSE	HR/%		
			±0.005%	±0.01%	±0.015%
SDC	0.0077	0.0112	53.71	82.23	95.45
SPA-H	0.0089	0.0125	49.73	79.50	92.86

## Data Availability

Data is unavailable due to privacy. Some of the data provided in this study are from third parties, and some are from our own research. All data has not been stored in the database.
